# Effectiveness of CT radiomic features combined with clinical factors in predicting prognosis in patients with limited-stage small cell lung cancer

**DOI:** 10.1186/s12885-024-11862-1

**Published:** 2024-02-03

**Authors:** Jiehan Wu, Yuntao Zhou, Chang Xu, Chengwen Yang, Bingxin Liu, Lujun Zhao, Jiawei Song, Wei Wang, Yining Yang, Ningbo Liu

**Affiliations:** 1 Department of Radiation Oncology, Tianjin Medical University Cancer Institute & Hospital, National Clinical Research Center for Cancer, Tianjin’s Clinical Research Center for Cancer, Key Laboratory of Cancer Prevention and Therapy, Tianjin, 300060 China; 2 Langfang Health Vocational College, Siguang Road, Guangyang District, Langfang, 065000 Hebei China; 3 College of Arts and Sciences, Lehigh University, 27 Memorial Drive West, Bethlehem, PA 18015 USA; 4 Department of Oncology, the People’s Hospital of Ganyu District, Lianyungang, 222100 China; 5 The Department of Radiotherapy, Tianjin First Central Hospital, Tianjin, 300192 China; 6 Hetian District People’s Hospital, Hetian, 848000 Xinjiang China

**Keywords:** Small cell lung cancer, Radiomics, Prognosis, Texture analysis, Simulated positioning CT

## Abstract

**Background:**

The prognosis of SCLC is poor and difficult to predict. The aim of this study was to explore whether a model based on radiomics and clinical features could predict the prognosis of patients with limited-stage small cell lung cancer (LS-SCLC).

**Methods:**

Simulated positioning CT images and clinical features were retrospectively collected from 200 patients with histological diagnosis of LS-SCLC admitted between 2013 and 2021, which were randomly divided into the training (*n* = 140) and testing (*n* = 60) groups. Radiomics features were extracted from simulated positioning CT images, and the t-test and the least absolute shrinkage and selection operator (LASSO) were used to screen radiomics features. We then constructed radiomic score (RadScore) based on the filtered radiomics features. Clinical factors were analyzed using the Kaplan–Meier method. The Cox proportional hazards model was used for further analyses of possible prognostic features and clinical factors to build three models including a radiomic model, a clinical model, and a combined model including clinical factors and RadScore. When a model has prognostic predictive value (AUC > 0.7) in both train and test groups, a nomogram will be created. The performance of three models was evaluated using area under the receiver operating characteristic curve (AUC) and Kaplan–Meier analysis.

**Results:**

A total of 1037 features were extracted from simulated positioning CT images which were contrast enhanced CT of the chest. The combined model showed the best prediction, with very poor AUC for the radiomic model and the clinical model. The combined model of OS included 4 clinical features and RadScore, with AUCs of 0.71 and 0.70 in the training and test groups. The combined model of PFS included 4 clinical features and RadScore, with AUCs of 0.72 and 0.71 in the training and test groups. T stages, ProGRP and smoke status were the independent variables for OS in the combined model, whereas T stages, ProGRP and prophylactic cranial irradiation (PCI) were the independent factors for PFS. There was a statistically significant difference between the low- and high-risk groups in the combined model of OS (training group, *p* < 0.0001; testing group, *p* = 0.0269) and PFS (training group, *p* < 0.0001; testing group, *p* < 0.0001).

**Conclusion:**

Combined models involved RadScore and clinical factors can predict prognosis in LS-SCLC and show better performance than individual radiomics and clinical models.

**Supplementary Information:**

The online version contains supplementary material available at 10.1186/s12885-024-11862-1.

## Introduction

SCLC has a poor prognosis with early metastatic dissemination and rapid recurrence. Limited-stage small cell lung cancer (LS-SCLC) accounts for one-third of small cell lung cancer (SCLC) cases, which is defined as the exclusion of distant metastatic disease by an international association [1, 2]. Although SCLC is sensitive to chemoradiotherapy, its 5-year overall survival (OS) rate is only 25% for patients with LS-SCLC due to the highly aggressive nature of the disease [3]. However, prognostic prediction of SCLC has proven to be enormously challenging. Despite conducting myriad studies on prognostic prediction of LS-SCLC, we have been unable to identify viable clinical factors for prognosis, such as peripheral blood inflammatory markers [4], pleural effusion [5], immune factors [6], and molecular subtypes [7], as none of them yielded satisfactory predictive outcomes. Thus, it is imperative to identify new prognostic prediction methods to enable informed clinical decision-making.

Radiomics is a technique that statistically analyses medical images without the involvement of physicians, allowing for non-invasive assessment of the entire tumour. Radiomic features, shape and higher order image features, can be extracted from computed tomography (CT), magnetic resonance imaging, and positron emission tomography(PET). Nowadays, radiomics are trying to be used to estimate the relationship between clinical and histopathological information in many tumors, even to predict prognosis of malignancy. In non-small cell lung cancer (NSCLC), radiomics is employed to predict prognosis [8, 9], metastases [10, 11], histological subtypes [12], and expression of epidermal growth factor receptors [13] and partly accepted as a powerful method. According to Kothari et al. [14] the area under the receiver operating characteristic (ROC) curve (AUC), ranging from 0.69 to 0.96, was utilised in seven studies. To predict prognosis of lung cancer with chemoradiotherapy, a radiomics model developed for predicting locoregional failure in NSCLC had an AUC of 0.776 in the testing population [15].

But in SCLC radiomics is far from the level of studies on NSCLC. Now is utilised mostly to classify histological subtypes [16–20]. Few studies have used radiomics to predict prognosis [21], whereas some published studies have shown that radiomics alone is not effective for prognostic prediction. The purpose of our study was to construct an available prognostic prediction model for LS-SCLC, so that we examined radiomic features and combined the radiomics with clinical factors. Consider chemoradiotherapy as the standard treatment and the key role of radiotherapy in LS-SCLC, we tried simulated positioning CT as the images of radiomics, which acquired at the very beginning of the radiotherapy and might ensures accuracy of the process. To our knowledge, none of the radiomics in SCLC were analysed in the data of simulated positioning CT, and this study first used the simulated positioning CT to evaluate the effectiveness of radiomic features combined with clinical factors on the prediction of prognosis in patients with LS-SCLC for appropriate individualised therapy for these patients.

## Materials and methods

### Patient characteristics

This study retrospectively identified patients with LS-SCLC admitted to Tianjin Medical University Cancer Institute and Hospital between September 2013 and March 2021. The Ethic Committee of Tianjin Medical University Cancer Institute and Hospital approved this study. Informed consent was obtained from all study participants.

The inclusion criteria were as follows: (1) patients with pathological confirmation of SCLC based on histological examination, (2) patients with LS-SCLC determined by imaging, and (3) patients receiving CT-based thoracic radiotherapy. The exclusion criteria were as follows: (1) patients with no clinical factors and positioning CT images, (2) patients receiving radiotherapy before thoracic radiotherapy, and (3) patients not receiving 30 fractions in thoracic radiotherapy. A total of 200 patients were enrolled in this study and randomly divided into the training (140 patients) and testing (60 patients) groups. For clinical, radiomics and combined models of OS and PFS, each model was independently grouped randomly.

### Clinical factors

Clinical factors included sex, age at diagnosis, smoking status, Karnofsky Performance Status Scale (KPS) score, and tumor (T), nodal (N), and metastasis (M) stages, routine blood tests, serum tumor marker levels, and immunohistochemistry of the tumor cells. The chemotherapy regimen and the performance of prophylactic cranial irradiation (PCI) were considered. The results of the last routine blood tests and serum tumor marker level assessment before simulated positioning CT were also included in this study. For routine blood tests, red blood cell count, white blood cell count, platelet count, haemoglobin level, neutrophil (NE) count, and lymphocyte (LY) count were measured. The NE/LY ratio was calculated based on these results. Serum tumor markers neuron-specific enolase (NSE) and pro-gastrin-releasing peptide (Pro-GRP) were assessed. Regarding immunohistochemistry, chromogranin A, thyroid transcription factor-1, and Ki-67 were evaluated.

### Computed tomography imaging parameters

Positioning CT images is necessary for patients to be treated with radiotherapy. All patients underwent CT simulation positioning using a helical CT scanner (CT Brilliance; Philips Medical Systems, Best, Netherlands). All the images captured before radiotherapy had a matrix of 512 × 512 voxels. The CT parameters were set as follows: voltage, 120 kV; exposure time (the triggering technique: delay), 50 s; range of the tube current, 200–250 mAs; and slice thickness, 3.0 mm. The simulated positioning CT of the patients included in this study was enhanced CT. Contrast agent protocols (80–100 ml at a rate of 2.0–3.0 ml/s) were relatively constant throughout all scans.

### Radiomic features

**Fig. 1:**
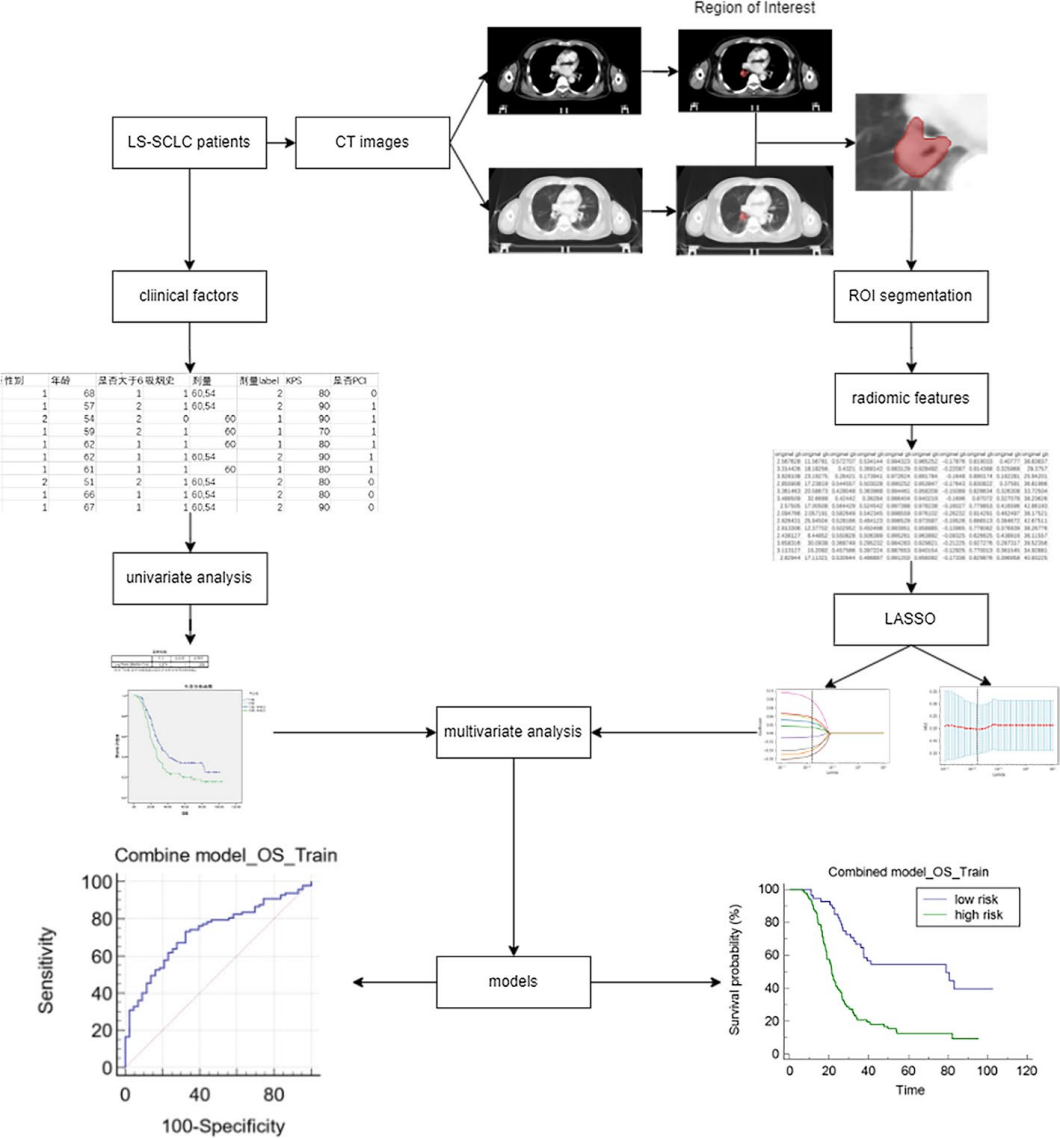
Workflow of the study with the ROC and Kaplan–Meier curve of the combined model in training group for OS

As shown in Fig. 1, all images were loaded into Pinnacle (3.2.0.27) to be predefined by two experienced radiation oncologists. The pinnacle was used to assess gross tumor volume (GTV), which was considered the manual region of interest (ROI), by two intermediate oncologists. The lung (–150 and –1150 HU) and mediastinal (215 and –135 HU) window levels were set to segment the tumor.

All ROIs were loaded into a three-dimensional slicer (4.11.20210226) to derive the features. Radiomic features were extracted using a radiomics package named “pyradiomics” that included first-order histogram, shape-based, and texture features. There are five types of texture features: the grey-level co-occurrence matrix (GLCM), grey-level size zone matrix (GLSZM), grey-level run-length matrix (GLRLM), neighbourhood grey tone difference matrix, and grey-level dependence matrix (GLDM). A three-dimensional wavelet transform function was used to calculate the wavelet characteristics. Applying low- (L) and high- (H) pass dimensional filters along three image axes resulted in eight deconstructed image sets: LLL-, LLH-, LHL-, LHH-, HLL-, HLH-, HHL-, and HHH-filtered images. We used Python (3.8.8) to apply the t-test and the least absolute shrinkage and selection operator (LASSO) to select radiomic features that had the strongest relevance with long-term survival.

### Statistical analyses

OS was calculated as the time from the date of diagnosis to the date of death from any cause. Progression-free survival (PFS) was calculated as the time from the start of radiotherapy to death or documentation of progression. In a cohort, the training and testing groups showed no statistical significance in the basic clinical data. Categorical variables were tested using the chi-squared test. The rank-sum test was used to compare the differences between continuous variables. Statistical significance was set at *p* < 0.05.

Clinical factors were estimated using the log-rank test. The Cox proportional hazards regression model included factors with *p*-values < 0.05 and radiomic features selected by LASSO. Radiomic features were normalized before they were selected by LASSO. In a cohort, there were three models: the clinical, radiomics, and combined (clinical factors and radiomic features) models. The performance of the models was evaluated by calculating the AUC, sensitivity, and specificity in the training and testing groups. The cut-off value calculated by the ROC curve was used to divide the high- and low-risk populations. Therefore, Kaplan–Meier survival analysis was performed to calculate survival differences between the high- and low-risk groups. The log-rank test and Kaplan–Meier survival analysis were performed using IBM SPSS Statistics version 23. The AUC was calculated using MedCalc version 20. The nomogram was drawn by R software(version 4.3.1; http://www.Rproject.org). Other statistical analyses were performed using scikit-learn, lifelines, pandas, and SciPy packages in Python 3.8.8.

## Results

### Clinical characteristics

As shown in Table 1, 152 males (median age, 59 [range, 23–79] years) and 48 females (median age, 61 [range, 31–81] years) were enrolled in this study. The median OS and PFS periods of all patients were 27.00 (range, 3.00–102.80) and 11.03 (range, 1.37–96.17) months, respectively, and the median follow-up period was 68.87 months. A total of 145 (72.5%) patients were smokers. In total, 57 (28.5%) patients were treated with 60 Gy in planning target volume (PTV) with radiotherapy, whereas the remaining patients (71.5%) were treated with 60 Gy in planning gross target volume and 54 Gy in PTV. In a cohort, the training group had no statistical significance with respect to basic clinical factors, including age, sex, smoking status, TNM stage, KPS score, and chemotherapy status.

**Table 1:** Clinical factors of LS-SCLC patients

Characteristic	Value	Percentage or Range	*P* value of OS	*P* value of PFS
**Sex**			0.859	0.704
Male	152	76.0%		
Female	48	24.0%		
**Age**	200	23–81(year)	0.051	0.147
**Smoking Status**			0.020	0.010
Yes	147	73.5%		
No	53	26.5%		
**KPS**			0.109	0.106
≤ 70	6	3.0%		
80	97	48.5%		
85	2	1.0%		
90	85	42.5%		
100	10	5.0%		
**T**			0.009	0.023
1	28	14.5%		
2	79	40.9%		
3	54	28.0%		
4	30	15.5%		
**N**			0.607	0.146
0	7	3.6%		
1	12	6.2%		
2	130	67.4%		
3	43	22.2%		
**M**				
0	200	100.0%		
1	0	0		
**Radiotherapy technology**			0.464	0.975
IMRT	139	69.5%		
VMAT	61	30.5%		
**Dose(Gy)**			< 0.001	< 0.001
GTV60	57	28.5%		
PGTV60,PTV 54	143	71.5%		
**Cycles of Neoadjuvant chemotherapy ≥ 2**			0.796	0.478
Yes	177	83.5%		
No	23	16.5%		
**Concurrent chemotherapy**			0.215	0.224
Yes	117	58.5%		
No	83	41.5%		
**Consolidation chemotherapy**			0.205	0.094
Yes	116	58.0%		
No	84	42.0%		
**Pro-GRP**	200	10.8–5000	< 0.001	< 0.001

### Radiomics feature selection

**Fig. 2:**
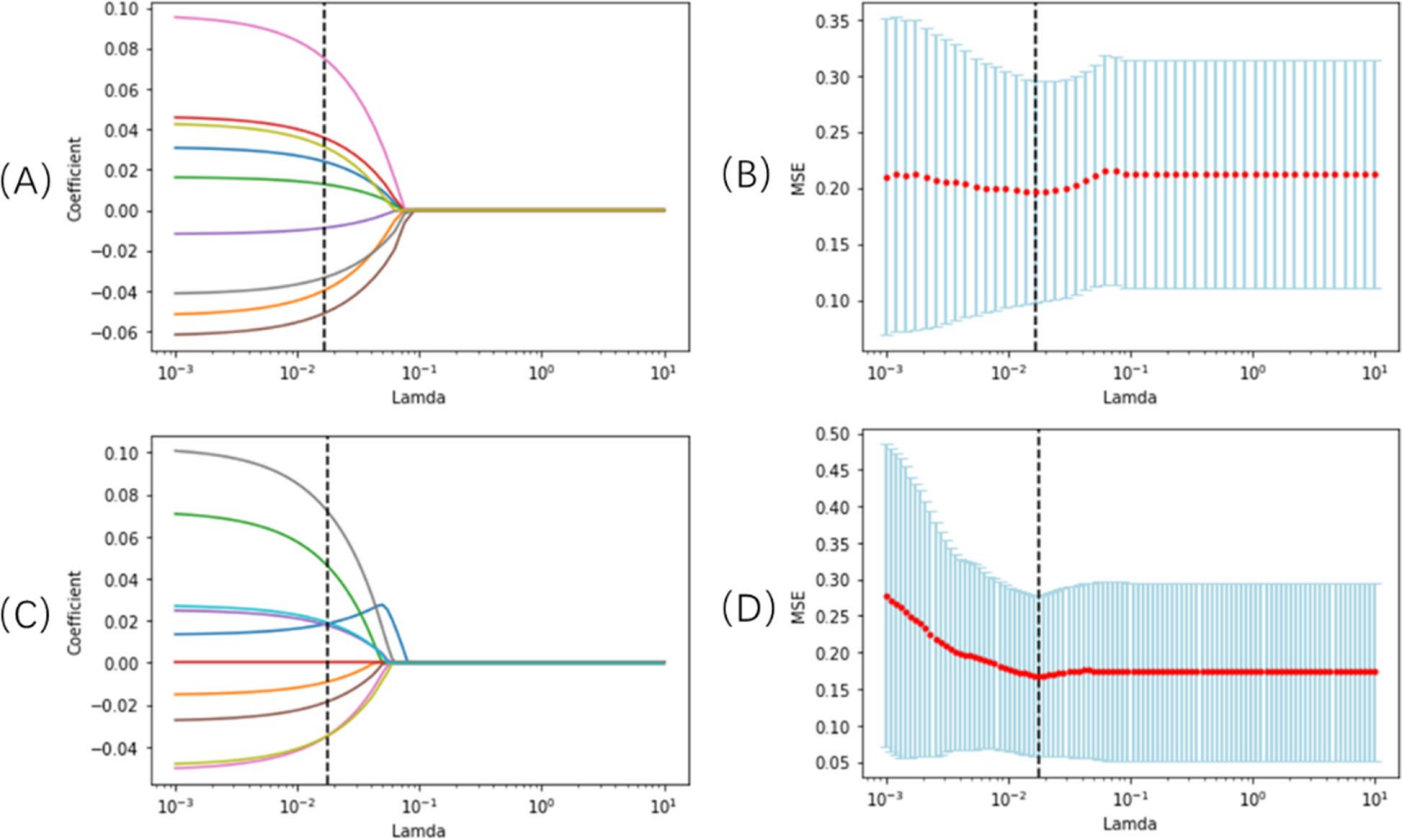
Selection of radiomic features using the least absolute shrinkage and selection operator model. **A** LASSO coefficient profiles of the 9 features of OS. **A** coefficient profile plot was produced against the log(lamda) sequence. **B** Selection of tuning parameter lamda in the LASSO regression using tenfold cross-validation via minimun criteria. Selection for overall survival with λ = 2.0236 × 10^–2^ (**C**) LASSO coefficient profiles of the 10 features of PFS. **D** Selection for progression-free survival with λ = 1.6768 × 10.^–2^

Patients were divided into the training and testing groups with the ratio 7:3. In total, 1037 features (Supplemental Fig. 1) were extracted from simulated positioning CT images. Nine radiomic features were selected based on LASSO for OS analysis (Fig. 2A, B), including one feature of shape and GLDM, two features of first order and GLSZM, and three features of GLCM. There were 10 radiomic features that were selected to construct the model of PFS analysis (Fig. 2C, D) combined with one feature of shape and GLRLM, two features of first order and GLSZM, and four features of GLCM. However, log-sigma-4–0-mm-3D_glcm_JointAverage and log-sigma-4–0-mm-3D_glcm_SumAverage have a multiplicative relationship; thus, only log-sigma-4–0-mm-3D_glcm_JointAverage was selected for analysis.

### Models for OS

Based on selected features for OS analysis, a radiomics model was established, named OS_R. Smoking status, PCI, T stage, and Pro-GRP analysed using the Kaplan–Meier method were included in the clinical model of OS (OS_C). The combined model of OS covered radiomic score (RadScore)_OS (the formula for RadScore_OS is shown in the Supplementary file) and all factors of clinical models of OS, named OS_RC. All models were created using the Cox proportional hazards regression model.

$$OS\_R=0.02\times logsigma50mm3D\_glszm\_GLNU+1.98\times logsigma50mm3D\_glszm\_SZNUN-0.01\times wavelet-LLH\_glcm\_ClusterShade-1.68\times wavelet-LLH\_glcm\_MCC+0.06\times wavelet-LHL\_firstorder\_Skewness+3.51\times wavelet-LHH\_glcm\_Correlation$$

$$OS\_C=0.23\times smoking status-0.54\times PCI+0.17\times T+0.00\times Pro-GRP$$

$$OS\_RC=0.69\times smoking \,status-0.36\times PCI+0.40\times T+0.00\times Pro-GRP+0.08\times RadScore$$

### Models for PFS

A Cox proportional hazards regression model was used to construct the models for PFS. The radiomics, clinical, and combined models were named as PFS_R, PFS_C, and PFS_RC, respectively. The clinical model covered four factors: smoking status, PCI, Pro-GRP and T stage, and the radiomics model covered nine features. The combined model of PFS covered RadScore_PFS (the formula for RadScore_PFS is shown in the Supplementary file) and all factors in the clinical model.

$$PFS\_R= 0.01\times original\_shape\_Maximum2DDiameterSlice+0.03\times logsigma40mm3D\_glcm\_Contrast +2.27\times logsigma50mm3D\_glszm\_SZNUN+0.01\times wavelet-LLH\_firstorder\_90Percentile-0.44\times wavelet-LLH\_firstorder\_Skewness+4.03\times wavelet-LLH\_glszm\_SAE-1.34\times wavelet-HHH\_glrlm\_RV$$

$$PFS\_C = 0.52\times smoking \,status-0.54\times PCI+0.26\times T+0.00\times Pro-GRP$$

$$PFS\_RC=0.43\times smoking \,stastus-0.59\times PCI+0.28\times T+0.00\times Pro-GRP-0.30\times RadScore$$

### Model evaluation

**Fig. 3:**
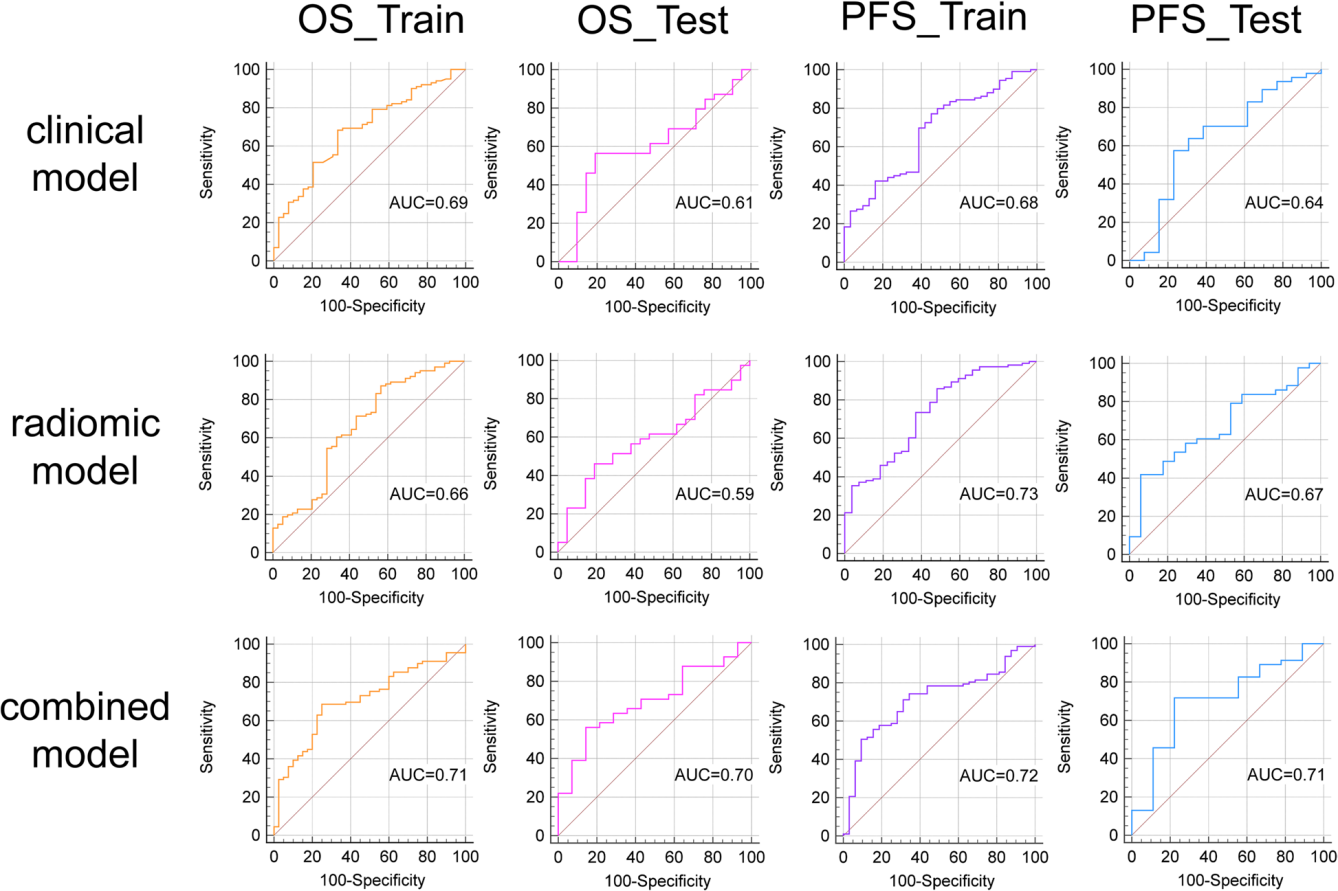
Receiver operating characteristic (ROC) curve of clinical models, radiomic models and combined models

**Fig. 4:**
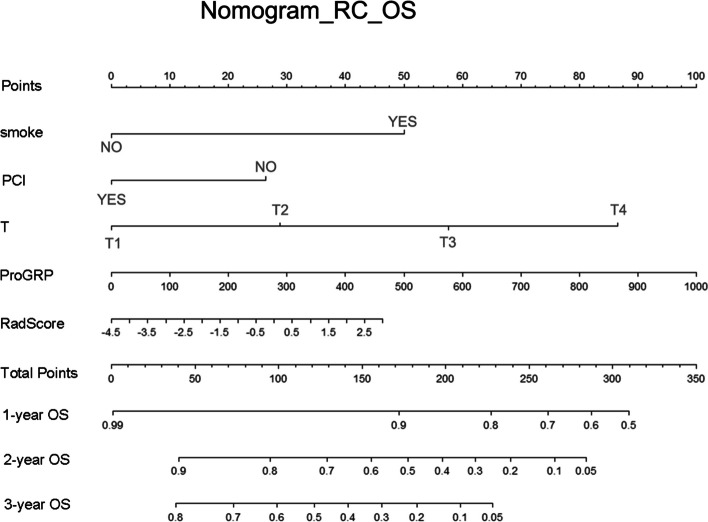
The nomogram of the combined model for predicting OS in patients with NSCLC

**Fig. 5:**
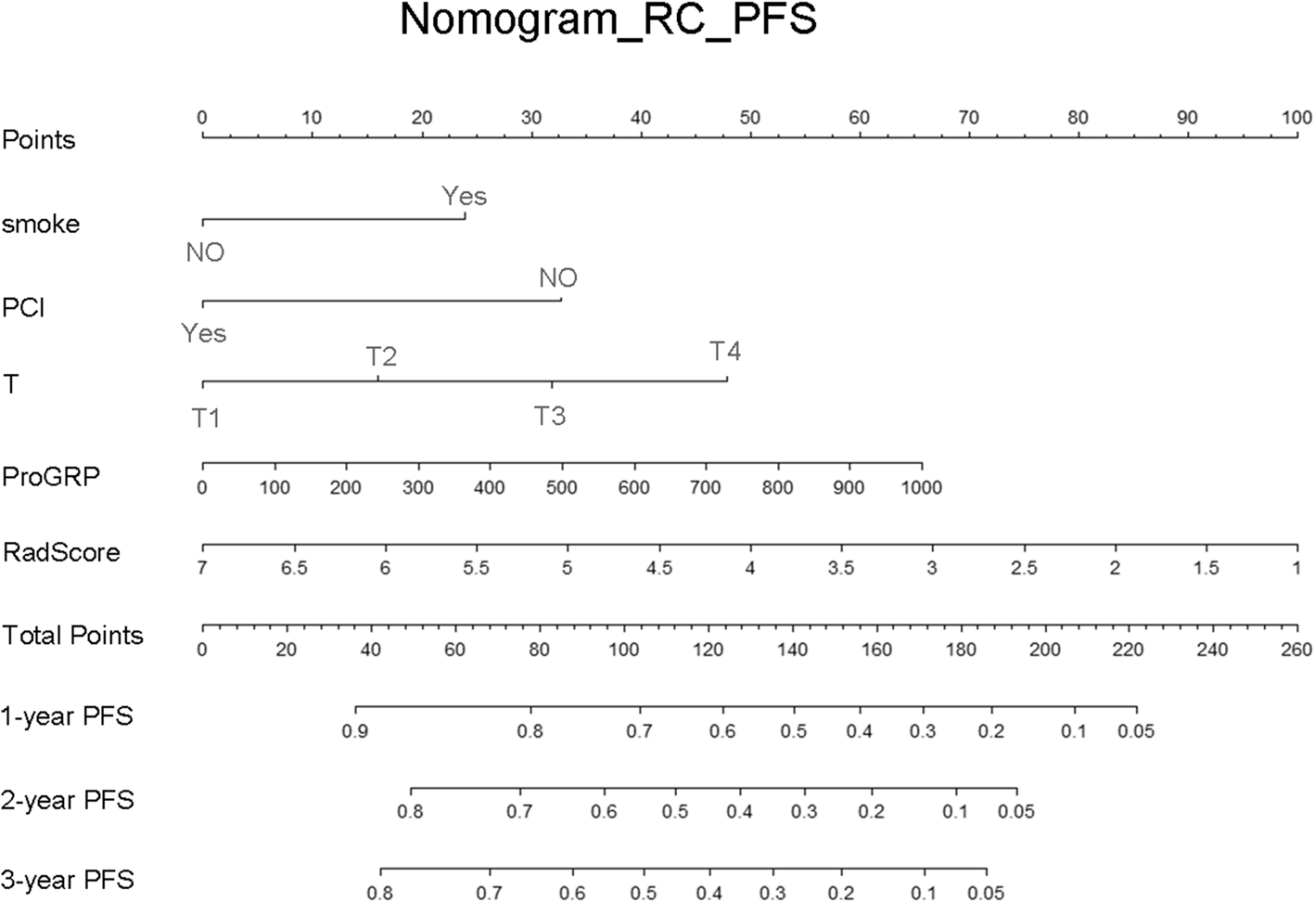
The nomogram of the combined model for predicting PFS in patients with NSCLC

**Fig. 6:**
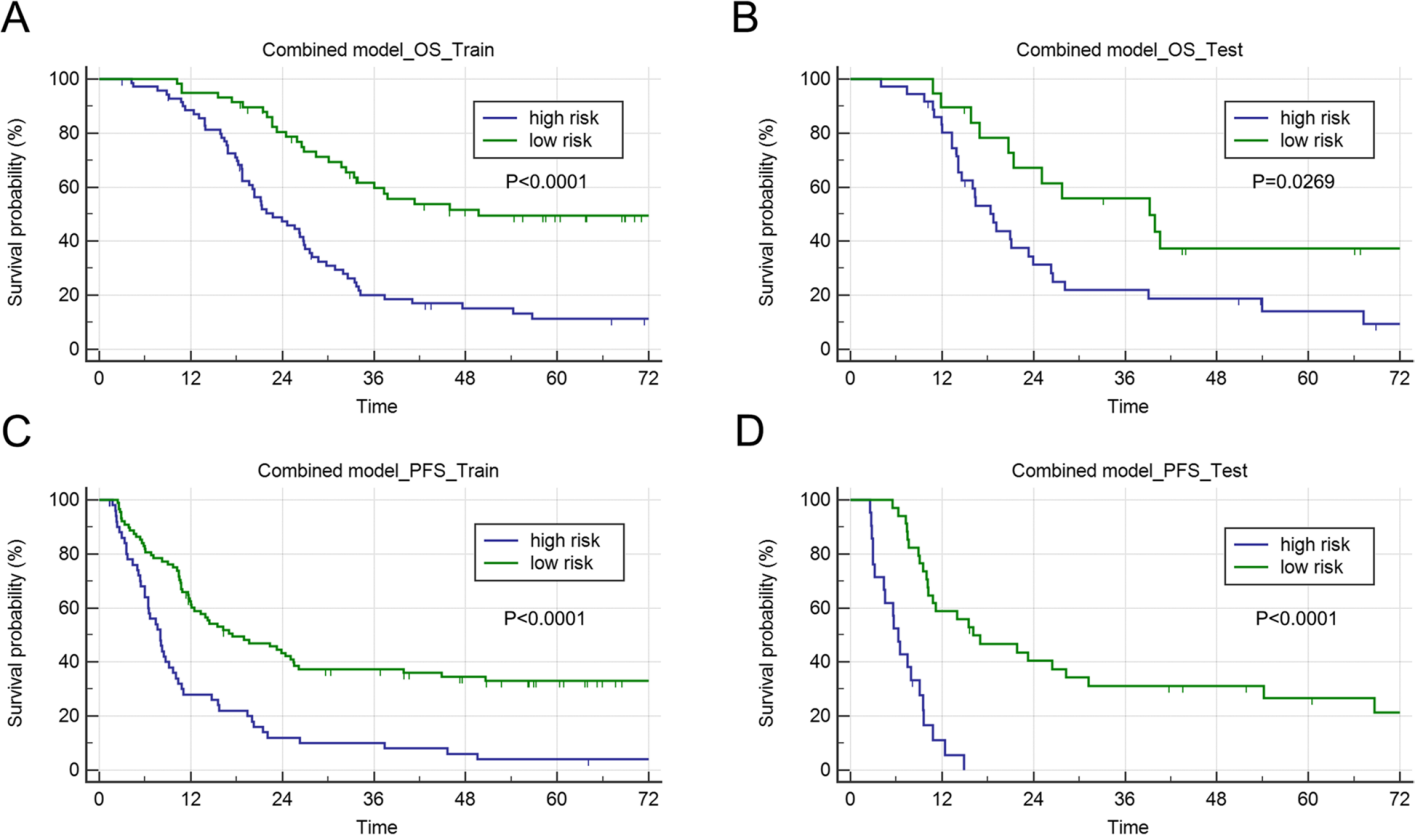
Kaplan–Meier curves of prognostic models to divide the high- and low-risk groups. **A** Kaplan-Meyer curve for the combined model of OS in the training group. **B** Kaplan-Meyer curve for the combined model of OS in the testing group. **C** Kaplan-Meyer curve for the combined model of PFS in the training group. **D** Kaplan-Meyer curve for the combined model of PFS in the testing group

In both the training and testing groups, models with AUCs > 0.7 were observed. The combined model (AUC of the training group, 0.71; AUC of the testing group, 0.70) of OS and the combined model (AUC of the training group, 0.72; AUC of the testing group, 0.71) of PFS (Table 2) were accessible. ROC curves of clinical models, radiomic models and combined models were showed in Fig. 3. ROC curves of the available models were used to calculate the cut-off value in the training group. OS_RC (cut-off = 1.57) and PFS_RC (cut-off = 0.31) were classified as low and high risk, respectively. The nomogram of the combined model for predicting OS and PFS in patients with NSCLC were shown in Figs. 4 and 5. According to the Kaplan–Meier survival analysis, there was a statistically significant difference between the low- and high-risk groups in the combined models of OS (training group, *p* < 0.0001; testing group, *p* = 0.0269) and PFS (training group, *p* < 0.0001; testing group, *p* < 0.0001) (Fig. 6). Comparison of cases with good and poor prediction results of OS and PFS was showed in Supplemental Fig. 2.

**Table 2:** AUC of predicted models

Characteristic		AUC	95%CI	Sensitivity (%)	Specificity (%)
OS_R	Train(*n* = 140)	0.66	0.57–0.74	87.13	43.59
	Test(*n* = 60)	0.59	0.46–0.72	46.15	80.95
OS_C	Train(*n* = 140)	0.69	0.60–0.76	68.32	66.67
	Test(*n* = 60)	0.61	0.47–0.73	56.41	80.95
OS_RC	Train(*n* = 140)	0.71	0.62–0.78	68.54	75.00
	Test(*n* = 60)	0.70	0.56–0.81	56.10	85.70
PFS_R	Train(*n* = 140)	0.73	0.65–0.81	85.84	51.85
	Test(*n* = 60)	0.67	0.54–0.79	41.86	94.12
PFS_C	Train(*n* = 140)	0.68	0.59–0.75	77.06	54.84
	Test(*n* = 60)	0.64	0.50–0.76	57.45	76.92
PFS_RC	Train(*n* = 140)	0.74	0.63–0.79	50.52	90.63
	Test(*n* = 60)	0.72	0.57–0.82	71.74	77.78

*Abbreviations*: *OS_R* radiomic model of OS, *OS_C* clinical model of OS, *OS_RC* combined model (including radiomic features and clinical factors) of OS, *PFS_R* radiomic model of PFS, *PFS_C* clinical model of PFS, *PFS_RC* combined model (including radiomic features and clinical factors) of PFS

## Discussion

Radiomics, which is a non-invasive and low-cost analysis with the use of machine learning, can portray information by extracting many features from images and can be utilised to predict histological subtypes and prognoses because texture features can present the heterogeneity of a tumor [22, 23]. SCLCs are highly aggressive and malignant which lead to poor prognosis and hard to predict prognosis, even in LS-SCLC. Our research group has conducted numerous studies on the timing of radiotherapy [24], the target area of radiotherapy [25, 26], radiotherapy techniques [27], treatment sequencing [28] and the number of cycles of chemotherapy [29] during chemoradiotherapy, as well as prophylactic cranial irradiation(PCI) [30] for SCLC. Our findings reveal the difficulty in accurately predicting the prognosis of LS-SCLC from a purely clinical standpoint. Therefore, there come to a strong need to find a method to predict the prognosis of SCLC.

Radiomics combined with clinical factors might be a powerful method to classify SCLC, and further as a powerful tool in the prediction of SCLC. In this study, OS and PFS predictive models were established based on clinical factors and radiomic features extracted from simulated positioning CT images of patients with SCLC.

Few studies have examined radiomics for SCLC prognostic prediction, and the minimal available studies have largely focused on classifying histological subtypes. Some published articles did not distinguish between limited and extensive stages, and the total number of cases was small. Gkika et al. [21]analysed 47 LS-SCLC patients and 51 extensive-stage (ES)-SCLC patients to evaluate immunohistochemical and radiomic features in predicting the survival time and did not present effective models. Another study proved that the radiomic features computed from tumor ROIs on both lung window and mediastinal window can predict the PFS for patients with SCLC by a high accuracy [31]. But the data were included not only 47 LS-SCLC patients but also 83 ES-SCLC patients. The number of LS-SCLC cases in each of the above studies was just over 40, a relatively small number of cases, accounting for no more than 50% of the total cases. Therefore, the results of the study are not representative of the effectiveness of radiomics in predicting the prognosis of LS-SCLC. In comparison, to our knowledge, we have established the largest database of radiomic analyses for prognostic prediction in LS-SCLC, which involved more patients, created available models, and presented more reliable results and more representative. The 2- and 5-year OS rates in our patients were 58.1% and 27.8%, respectively. The median OS was 27.00 months which was similar to the other study [32], which provided an powerful data to further evaluate the prognosis of our work.

In this study, 1037 features were extracted based on the GTV and involved first-order histogram, shape-based, texture, and wavelet features, which to our knowledge is the most features extracted among any similar study. The results are more trustworthy because more radiomic features were extracted. A previous study based on SCLC [21]extracted 72 texture features; hence, the findings of this study had a medium level of confidence. Some studies have also included a wider variety of features, including first-order, shape, high-order texture, and wavelet features; however, the number of extracted features was only 680 [33]. Compared with previous studies, our data was based on machine learning, which extracts a greater number and variety of features and provides more accurate predictions of prognosis.

According to NCCN guidelines, chemoradiotherapy is the standard of care for patients with LS-SCLC [34]. Accordingly, a survey of 206 Chinese oncologists revealed unanimous physician endorsement of thoracic radiotherapy for LS-SCLC patients [35], demonstrating the widespread use of radiotherapy in the treatment of LS-SCLC. Normally, radiomic features are extracted from images that are used to diagnose diseases at a higher resolution [22, 23] and collect more quantitative data than the naked eye and help with clinical judgment. Our study innovatively used simulated positioning CT images before radiotherapy for radiomics analysis. Compared to the high resolution diagnostic CT at the initial diagnosis of LS-SCLC, simulated positioning CT images used in our study were much closer to the pre-treatment situation, which were more credible to radiomics. Consider the key treatment role of radiotherapy in LS-SCLC, simulated positioning CT images were also easy to obtain. In addition, the direct use of GTV as ROIs eliminates the need to outline ROIs compared to classical radiomics; at the same time, the ROIs delineated by the radiation oncologists is more accuracy in the definition of the tumor mass which will be irradiated in the LS-SCLC patients. In conclusion, simulated positioning CT has unique advantages in radiomics applications, and we also believe that it will be used more and more in radiomics.

In our study, good performance was not observed in either OS (AUC of the training group, 0.66; AUC of the testing group, 0.59) or PFS (AUC of the training group, 0.73; AUC of the testing group, 0.67) as the standard of AUCs ≥ 0.7, based on radiomics models alone, which was similar to the other studies. Original_shape_Maximum2DDiameterColumn, original_glcm_Imc1, and original_glcm_Imc2 were selected to construct models with OS and PFS using the Cox proportional hazards regression model as bivariate models in a previous study [21]. The feature that had the strongest correlation with OS/PFS was constructed bivariate with NSE and lactate dehydrogenase, but the models were not able to predict survival. The models developed by Chen et al. [33] to predict PFS using radiomic features had a mean AUC of 0.8487, but ES-SCLC and LS-SCLC were included in the same work, and no stratified analysis was performed, which led to a reduction in the credibility of the study in terms of LS-SCLC. In contrast, a more reliable result was obtained in our study based on radiomics models with only patients with LS-SCLC. The poor performance of our radiomics model showed the highly malignant and diverse behaviours of the SCLC.

Ki-67, CgA, and TTF-1 were included for analysis regarding to immunohistochemistry. The Ki-67 index is recognised as a reliable predictor of the prognosis of various types of tumours, including SCLC. A study by Ding et al. highlighted that Ki-67 could serve as an independent prognostic factor for SCLC patients who have undergone surgery [36]. Additionally, research conducted by Böhm et al. indicated a negative correlation between Ki-67 expression and the survival rate of SCLC patients [37]. Meanwhile, the expression of CgA and TTF-1 in SCLC also possesses prognostic predictive value. Hamanaka's study demonstrated that patients negative for CgA had a better prognosis [38]. Similarly, Petrović et al.'s study found that CgA potentially plays a role in predicting the prognosis of SCLC patients [39]. Wang et al.'s meta-analysis indicates that TTF-1 possibly acts as an autonomous prognostic marker among SCLC patients [40]. There is no significant distinction in overall survival between TTF-1 negative and TTF-1 positive SCLC patients as well [41]. Therefore, we examined the prognostic potential of these three immunohistochemical factors as clinical factors for SCLC prognosis. However, they failed the log-rank test in this model and were thus excluded from our predictive model.

It is difficult to predict the clinical prognosis of patients with SCLC in a long run. Clinical models were constructed in our study and there were still no available models to be constructed for OS (AUC of the training group, 0.69; AUC of the testing group, 0.61) or PFS (AUC of the training group, 0.68; AUC of the testing group, 0.64). This might due to the small number of patients, which prevented the fitting of a model with high predictive efficacy, or due to the heterogeneity and malignant behavior of the SCLC.

The combined models showed the best performance in our study, with AUCs higher than those of radiomics and clinical models separately. This aligns with the favorable prognostic predictive ability of radiomics with clinical features in colorectal cancer [42], cervical cancer [43], esophageal cancer [22], and NSCLC [23]. These results showed that radiomic features combined with clinical factors were more available than single radiomic features and clinical factors for prognostic prediction. In the combined model of OS, T stages, ProGRP and smoke status were the independent factors, based on the results of the Cox proportional hazards regression model. Similar to the OS model, T stages, ProGRP and PCI were the independent factors of the combined PFS model. PCI has been shown to reduce brain metastasis and increase survival in patients with LS-SCLC who respond well to definitive chemoradiotherapy [44]. The metastatic spread of cancer to distant organs is the reason for most cancer-related deaths [45]. In addition, in this study, we established exclusion criteria, such as excluding patients who did not receive 30 divisions during chest radiotherapy. Our main aim was to ensure comparability of patients' baseline characteristics and to avoid the prognostic impact of different radiotherapy fractions on patients' prognosis. Therefore, we can assume that the proposed model is reliable. The Kaplan–Meier curve confirmed that the combined models effectively divided the high- and low-risk groups as the cut-off value calculated by AUC. There was a significant survival benefit in the low-risk group compared to that in the high-risk group. The validity of the models was demonstrated again.

This study has some limitations. First, we presented a retrospective study design at a single institution with a limited number of participants, so selection bias may inevitably exist. Second, our model lacks external validation. Finally, the radiological features were manually segmented by two radiation oncologists, which may be influenced by subjective trends of the observers. In the future, our objective is to construct a more extensive database for SCLC radiomics and construct multiple predictive models using different machine learning methods, and ultimately filter out the model with the best prognostic predictive efficacy. These models will then undergo external validation to enhance their practicality and reliability.

In conclusion, our retrospective study found that radiomics based on simulated positioning CT can be used to establish models to predict outcomes. LS-SCLC patients can be divided into the high- and low-risk groups according to our combined models which might further lead to individualised therapeutic decision-making in the near future.

### Supplementary Information

**Additional file 1: **The formula for RadScore_OS and RadScore_PFS.** Supplemental Figure 1. **Heatmap of all features. **Supplemental Figure 2.** Comparison of cases with good and poor prediction results.

## Data Availability

All data generated or analysed are included in this article.

## Abbreviations

AUC: Area under the receiver operating characteristic curve
CT: Computed tomography
ES-SCLC: Extensive-stage small cell lung cancer
GLCM: The grey-level co-occurrence matrix
GLDM: Grey-level dependence matrix
GLRLM: Grey-level run-length matrix
GLSZM: Grey-level size zone matrix
GTV: Gross tumor volume
H: High
KPS score: Karnofsky Performance Status Scale
L: Low
LASSO: The least absolute shrinkage and selection operator
LS-SCLC: Limited-stage small cell lung cancer
LY: Lymphocyte
OS: Overall survival
PCI: Prophylactic cranial irradiation
PTV: Planning target volume
PET: Positron emission tomography
PFS: Progression-free survival
ROC: The receiver operating characteristic
ROIs: Regions of interest
Pro-GRP: Pro-gastrin-releasing peptide
SCLC: Small cell lung cancer
NE: Neutrophil
NSCLC: Non-small cell lung cancer
NSE: Neuron-specific enolase
