# Supplementary material for: Effectiveness of CT radiomic features combined with clinical factors in predicting prognosis in patients with limited-stage small cell lung cancer
Source: BMC Cancer. 2024 Feb 3;24:170. doi: 10.1186/s12885-024-11862-1 (PMC10838455; doi:10.1186/s12885-024-11862-1)
Supplement: Supplementary file 1 — Additional file 1: The formula for RadScore_OS and RadScore_PFS. Supplemental Figure 1. Heatmap of all features. Supplemental Figure 2. Comparison of cases with good and poor prediction results. [file 12885_2024_11862_MOESM1_ESM.docx]

**The formula for RadScore_OS and RadScore_PFS**

$$RadScore\_OS=0.02\times logsigma50mm3D\_glszm\_GLNU+1.98\times logsigma50mm3D\_glszm\_SZNUN-0.01\times wavelet-LLH\_glcm\_ClusterShade-1.68\times wavelet-LLH\_glcm\_MCC+0.06\times wavelet-LHL\_firstorder\_Skewness+3.51\times wavelet-LHH\_glcm\_Correlation$$

$$RadScore\_PFS= 0.01\times original\_shape\_Maximum2DDiameterSlice+0.03\times logsigma40mm3D\_glcm\_Contrast +2.27\times logsigma50mm3D\_glszm\_SZNUN+0.01\times wavelet-LLH\_firstorder\_90Percentile-0.44\times wavelet-LLH\_firstorder\_Skewness+4.03\times wavelet-LLH\_glszm\_SAE-1.34\times wavelet-HHH\_glrlm\_RV$$

**Supplemental Figure Legends**

**Supplemental Figure 1.** Heatmap of all features.

**Supplemental Figure 2.** Comparison of cases with good and poor prediction results. (A) Images with good OS prediction results. (B) Images with poor OS prediction results. (C) Images with good PFS prediction results. (D) Images with poor PFS prediction results.


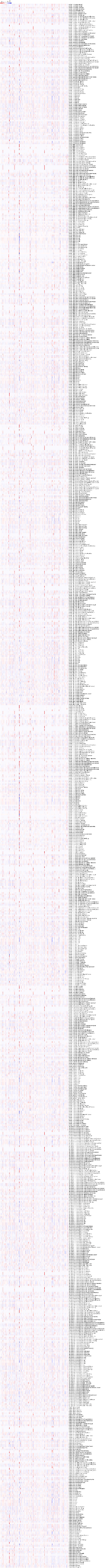


**Supplemental Figure 1.** Heatmap of all features.


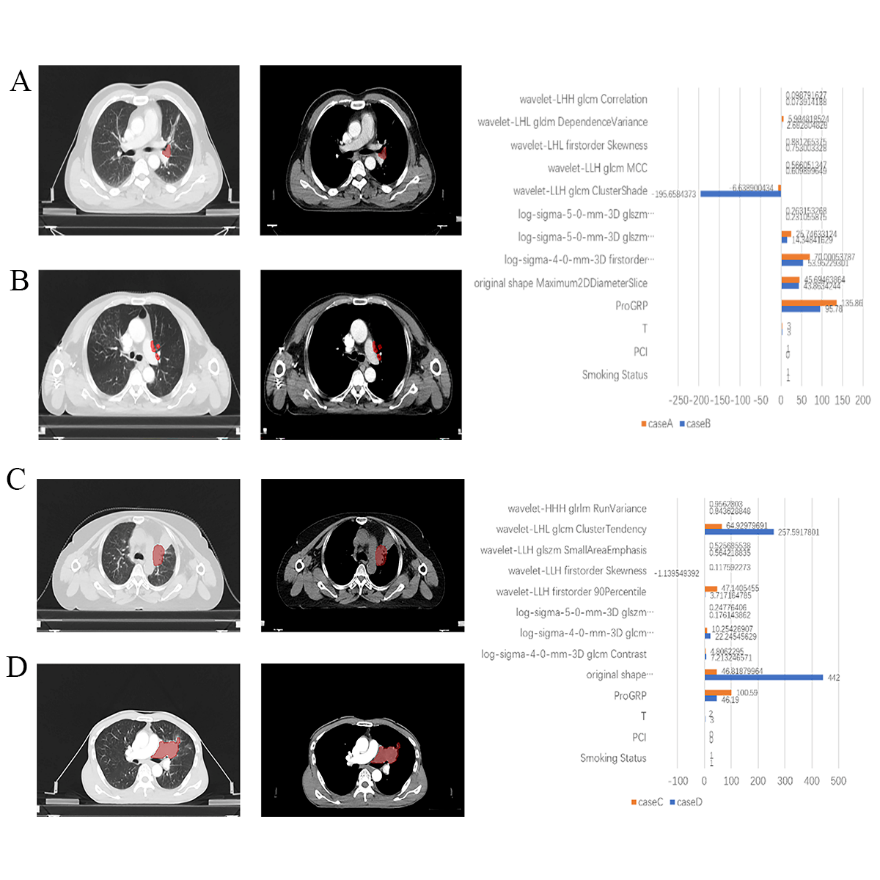


**Supplemental Figure 2.** Comparison of cases with good and poor prediction results. (A) Images with good OS prediction results. (B) Images with poor OS prediction results. (C) Images with good PFS prediction results. (D) Images with poor PFS prediction results.
